# Supplementary material for: Mesostats—A multiplexed, low-cost, do-it-yourself continuous culturing system for experimental evolution of mesocosms
Source: PLoS One. 2022 Jul 28;17(7):e0272052. doi: 10.1371/journal.pone.0272052 (PMC9333204; doi:10.1371/journal.pone.0272052)
Supplement: S1 File — (PDF) [file pone.0272052.s001.pdf]

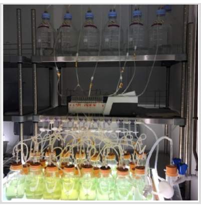

Mesostats --- A multiplexed, low-cost, do-it-yourself continuous culturing system for experimental evolution of mesocosms

Erika M Hansson[1], Dylan Z. Childs[1], Andrew P. Beckerman[1]

[1]The University of Sheffield, Sheffield, United Kingdom

1 Works for me

Erika Hansson

ABSTRACT

Microbial experimental evolution allows studying evolutionary dynamics in action and testing theory predictions in the lab. Experimental evolution in chemostats (i.e. continuous flow through cultures) has recently gained increased interest as it allows tighter control of selective pressures compared to static batch cultures, with a growing number of efforts to develop systems that are easier and cheaper to construct. This protocol describes the design and construction of a multiplexed chemostat array (dubbed "mesostats") designed for cultivation of algae in 16 concurrent populations, specifically intended for studying adaptation to herbicides.

PROTOCOL INFO

Erika M Hansson, Dylan Z. Childs, Andrew P. Beckerman . Mesostats --- A multiplexed, low-cost, do-it-yourself continuous culturing system for experimental evolution of mesocosms. **protocols.io**
https://protocols.io/view/mesostats-a-multiplexed-low-cost-do-it-yourself-co-ca86shze

CREATED

Jun 13, 2022

LAST MODIFIED

Jun 17, 2022

PROTOCOL INTEGER ID

64510

GUIDELINES

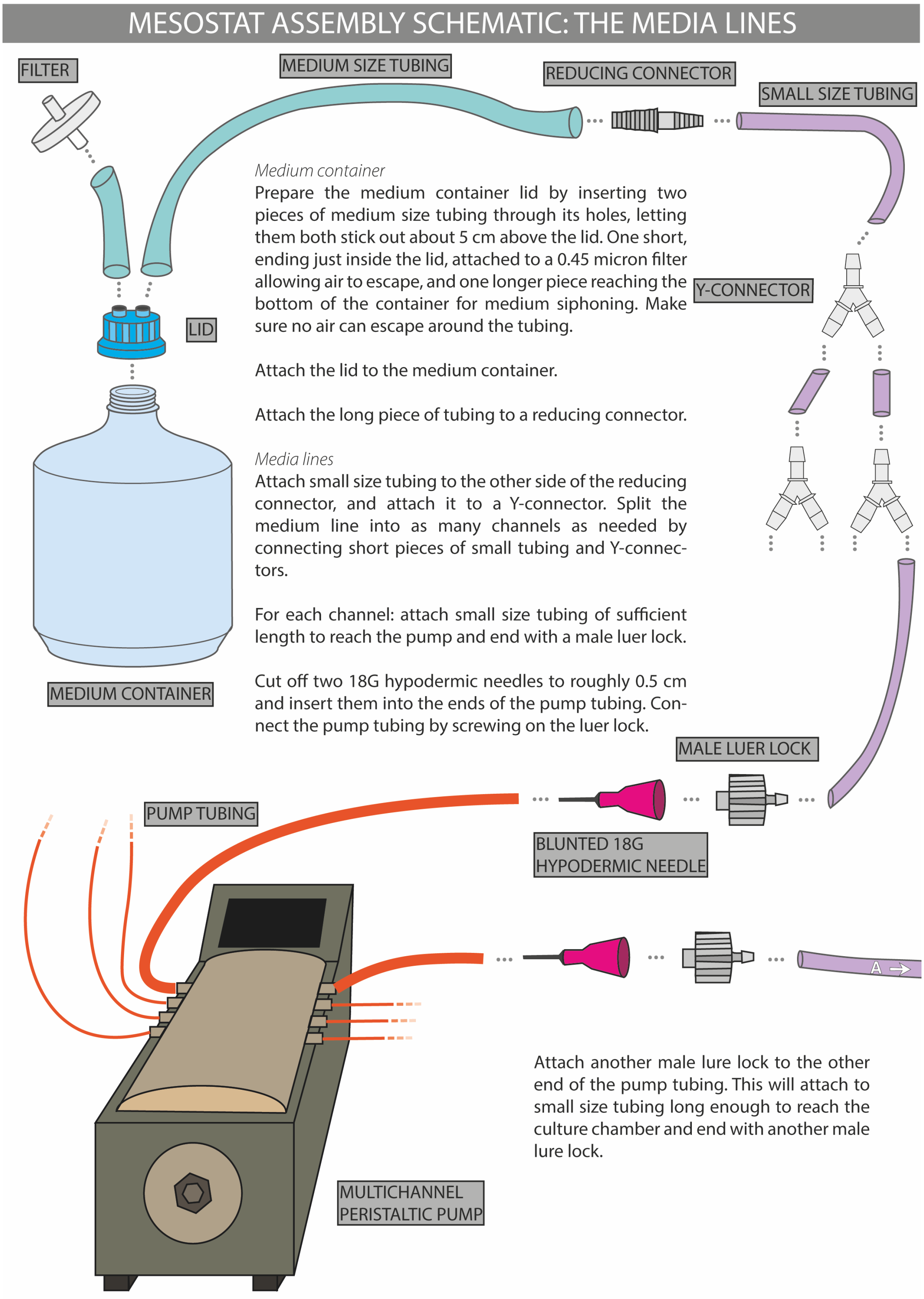

FIG 3: Assembly schematic; the media lines.

MESOSTAT ASSEMBLY SCHEMATIC: THE CHAMBERS

Culture chamber
Prepare the culture chamber rubber bung lid by drilling 1mm holes for each hypodermic needle. Push through one 18G short needle (medium influx), one 18G 20 cm needle (air influx) and two 18G 10 cm needles (culture efflux, sampling needle). Mark the glass jar at the desired culture volume.

Medium influx: Attach the media line to the medium influx needle.

Sampling needle: Cover with male luer lock cap.

Culture efflux: Attach a male luer lock and set needle at required height for desired culture volume.

Overflow chamber: Drill two holes in the overflow chamber rubber bung to fit two female luer locks. Connect one hole with small size tubing to the male luer lock on the culture efflux needle, and attach another female luer lock underneath as a funnel. Attach a short piece of small size tubing to the other hole and end with a 0.2 micron filter.

Air influx: Attach a male luer lock and push needle to the bottom. Connect a small size tube long enough to reach the 4-port manifold.

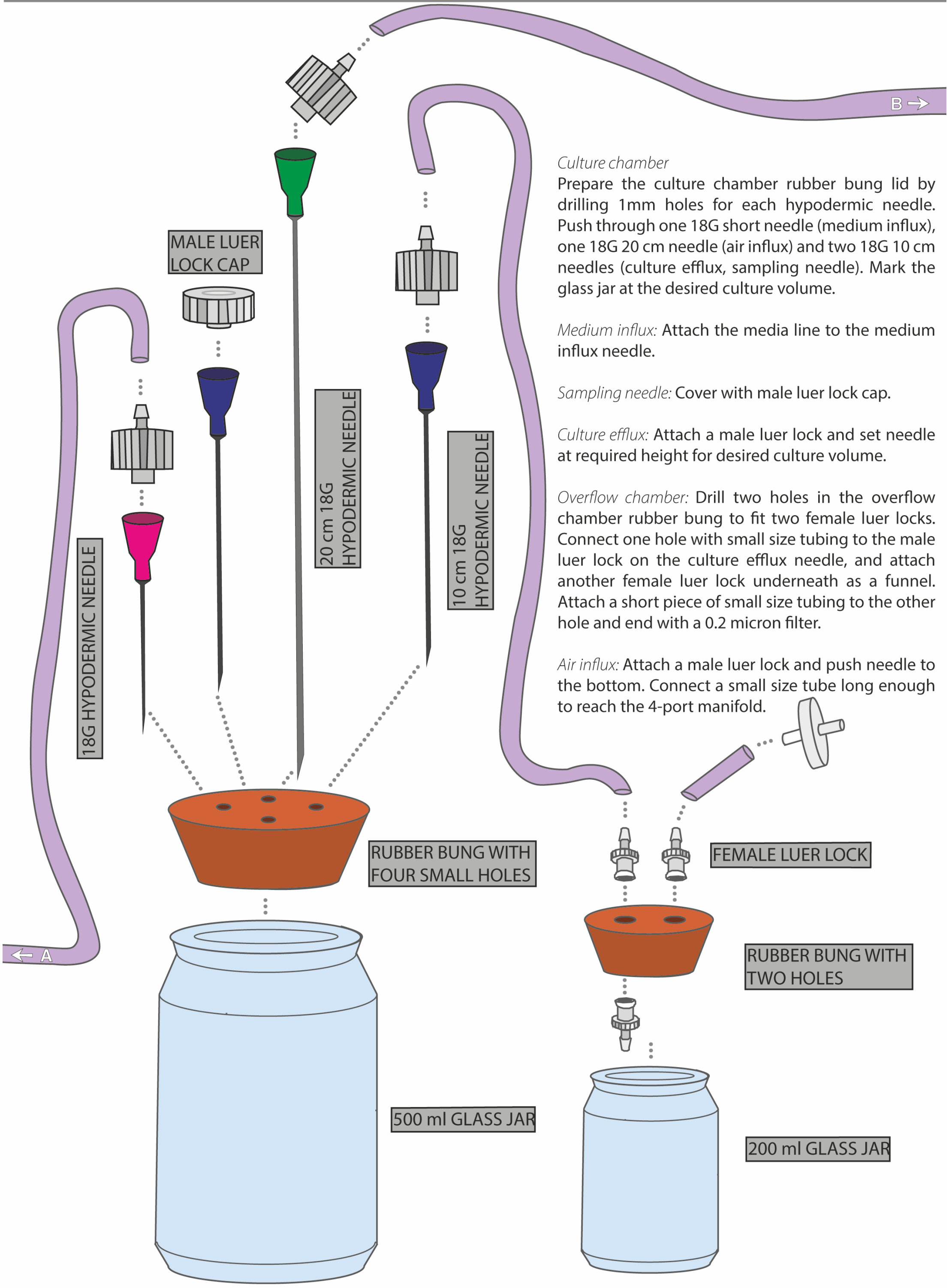

FIG 4: Assembly schematic; the chambers.

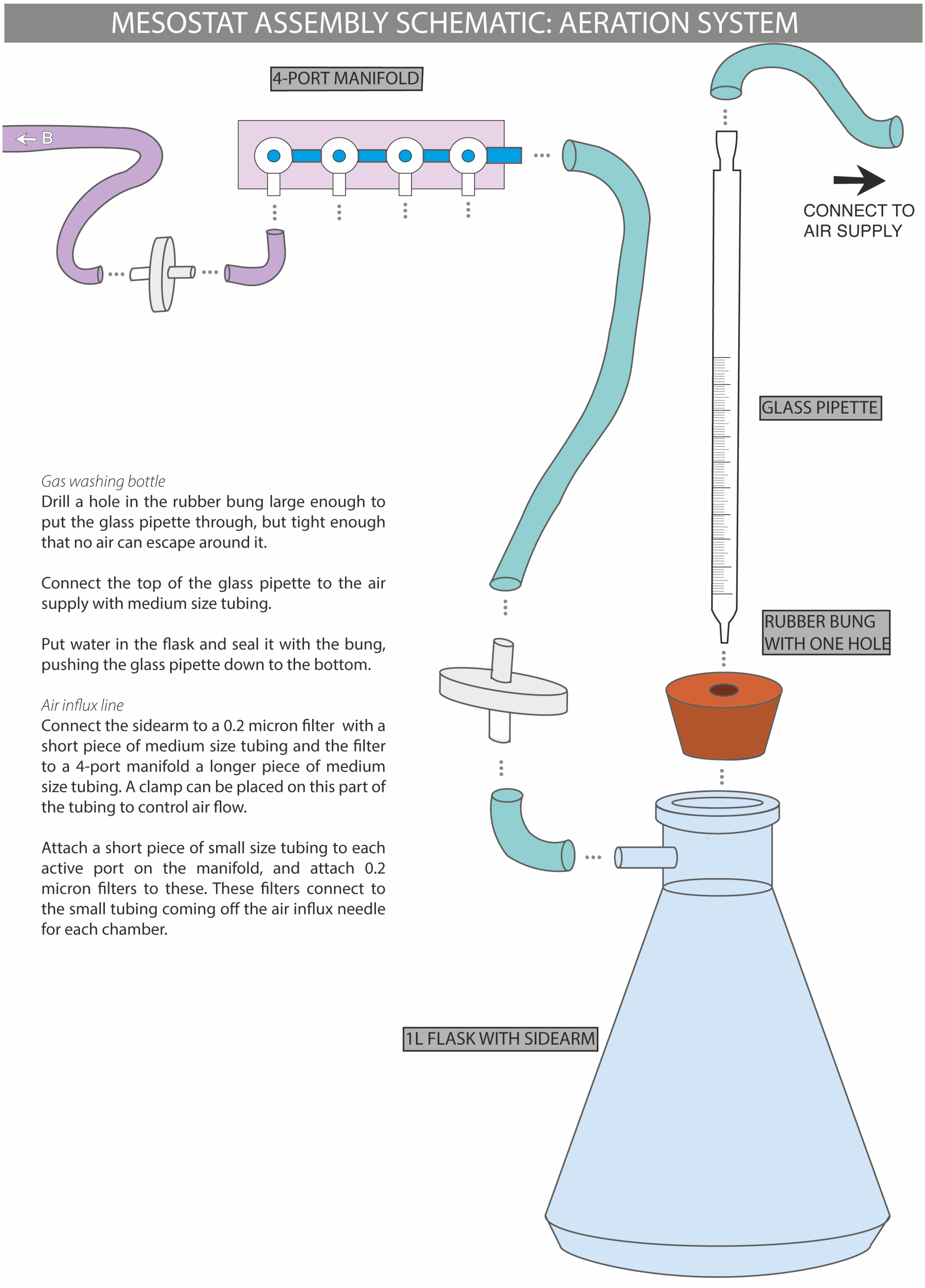

FIG 5: Assembly schematic; the aeration system.

MATERIALS TEXT

Here we present a complete list of materials required to construct a 16-chamber array. Note that the media containers and associated lids are listed as optional, as exact size and number needed depend on the experimental design. If the company and product code is not listed, the part was not acquired new and the exact same product is no longer sold. Other than the pump and pump tubing, all of the pieces are fairly standard pieces found in many wet labs and similar products can be obtained easily from all major scientific suppliers.

1. Small tubing, 20 m (Fisherbrand™ Silicone Tubes; inner/wall diameter: 3.0/1.0 mm, Fisher Scientific, 10111801)
2. Medium tubing, 20 m (Fisherbrand™ Silicone Tubes; inner/wall diameter: 6.5/1.5 mm, Fisher Scientific, 10549201)
3. Large tubing, 10 m (Fisherbrand™ Silicone Tubes; inner/wall diameter: 12.5/2.25 mm, Fisher Scientific, 10726931)
4. Reducing connector, 10 (Reducing Connector PVDF; 1/4" to 1/8", Cole Parmer, EW-30703-50)
5. Male luer lock, 100 (Cole-Parmer ADCF Male Luer to 1/8" L Barb Adapter, Cole Parmer, WZ-30800-24)
6. Female luer lock, 50(Cole-Parmer ADCF Female Luer to 1/8" L Barb Adapter, Cole Parmer, WZ-30800-08)
7. Y-connector, 40 (Barbed Y Connector; PVDF; 1/8",Cole Parmer, WZ-30633-44)
8. Straight connector, 10 (Barbed fittings; Straight Connector; Kynar; 1/4" ID, Cole Parmer, WZ-30703-05)
9. Gas washing bottle, 1 (1L flask with sidearm tubulation)
10. Glass pipette, 1 (10 ml glass pipette)
11. 4-port Manifold, 4 (Polycarbonate individual manifolds with luer locks; 4 ports; 180° rotation, Cole Parmer, EW-06464-85)
12. 0.45 µm filters, 100 (PTFE Nonsterile Syringe Filters; 0.45 micron; 25 mm dia, Cole Parmer, WZ-02915-22)
13. 0.2 µm filters, 1 (AcroVent 0.2µm PTFE, Pall Corporation, 4249)
14. Air tubing clamp, 1 (Adjustable tubing clamp)
15. Multiplexed peristaltic pump, 1 (205S/CA16 16 Cartridge pump, Watson-Marlow, 020.3716.00A)
16. Pump tubing, 18 (Autoclaveable marprene manifold pump tubing; orange/orange; 0.88 mm bore, Watson-Marlow, 978.0088.00+)
17. Culture chamber jars, 16 (Clear glass powder jars; 500 ml)
18. Culture chamber rubber bungs, 16 (Fisherbrand™ Solid Rubber Stoppers; 45 mm bottom; 51 mm top, Fisher Scientific, 41122502)
19. 16 (Central Surgical Company™Stainless Steel Needle; 16 G; 203 mm, Fisher Scientific, 12329259)
20. Media influx needle, 48 (B Braun™ Hypodermic Needles Pink 1.2 mm 18 G 50 mm, Fisher Scientific, 10722784)
21. Efflux needle, 16 (Central Surgical Company™ Stainless Steel Needle; 16 G; 101 mm, Fischer Scientific, 12339259)
22. Sampling needle, 16 (Central Surgical Company™ Stainless Steel Needle; 16 G; 101 mm, Fischer Scientific, 12339259 \textit{or} Central Surgical Company™ Stainless Steel Needle; 16 G; 203 mm, Fisher Scientific, 12329259)
23. Male luer cap, 16 (Male Luer Lock Plug; Nylon, Cole Parmer, WZ-45505-56)
24. Collection chamber jars, 32 (Clear glass powder jars; 175 ml)
25. Collection chamber rubber bungs, 16 (Fisherbrand™ Solid Rubber Stoppers; 37 mm bottom; 42.5 mm top, Fisher Scientific, 41122502)
26. 20 L medium container, 2 *optional*
27. Silicone stopper for 20 L medium container, 2 *optional*
28. 5 L medium container, 2 (Pyrex™ Borosilicate Glass Reagent Bottles with Polypropylene Cap and Pouring Ring; 5000 mL, Fisher Scientific, 12094637) *optional*
29. 2 L medium container, 20 (Pyrex™ Borosilicate Glass Reagent Bottles with Polypropylene Cap and Pouring Ring; 2000 mL, Fisher Scientific, 11922629) *optional*
30. Lids with holes for 5 L and 2 L medium containers,14 (GL45 Screw cap for Pyrex GL 45 media-lab bottle, Fisher Scientific, 15173927) *optional*
31. Syringes, 100 (BD Discardit™ Eccentric Luer-Slip Two-Piece Syringe, Fisher Scientific,

10152534)
32. Linear LEDs (LEDVANCE, 600 10 W, 3000 K warm white)

BEFORE STARTING

An overview of the design

The mesostat array consists of four main parts: (1) the medium line, (2) the culture chambers, (3) the overflow chambers and the (4) aeration line (Fig 1). Medium is pumped from the medium containers via a peristaltic pump into the culture chambers (mesocosms) where the experimental organisms are grown. Air is pumped through a gas washing bottle into the culture chambers via the aeration needle to ensure mixing and create pressure so liquid flows out through the efflux needle and the culture stays a fixed volume. The efflux line leads to an overflow chamber, and the volume collected in these is regularly measured to ensure equal flow rates between all culture chambers. Samples for analysis can be obtained from the overflow, but this only samples from the top of the culture and the environment in the collection receptacle may differ from the culture chambers. To allow sampling from lower levels of the culture, sampling needles have been fitted to the culture chambers. The sampling needle can also be used for inoculation or addition of treatments to the chambers. The entire setup can be kept in a controlled temperature room which ensures low levels of evaporation, but the culture temperature can also be maintained by other methods such as a light table or a water bath. When growing photosynthetic organisms such as algae, even light levels for all chambers are best maintained by a light table as well as fitting strip lights around the chambers. An overview of the full design is seen in Fig 1 and Fig 2A. The control conditions for *Chlamydomonas reinhardtii* cultures are summarised in Table 1.

A	B
**Light level**	75 µmol/m2/s, 24 h, all directions
**Internal culture temperature**	30°C
**CT room ambient temperature**	25°C
**Medium flow rate**	0.15/day (1.25 RPM pump speed)
**Sampling frequency and volume**	1.5 ml/day
**Medium**	Ebert algal medium (Ebert, 2013)

Table 1: Control conditions for *C. reinhardtii* cultures.

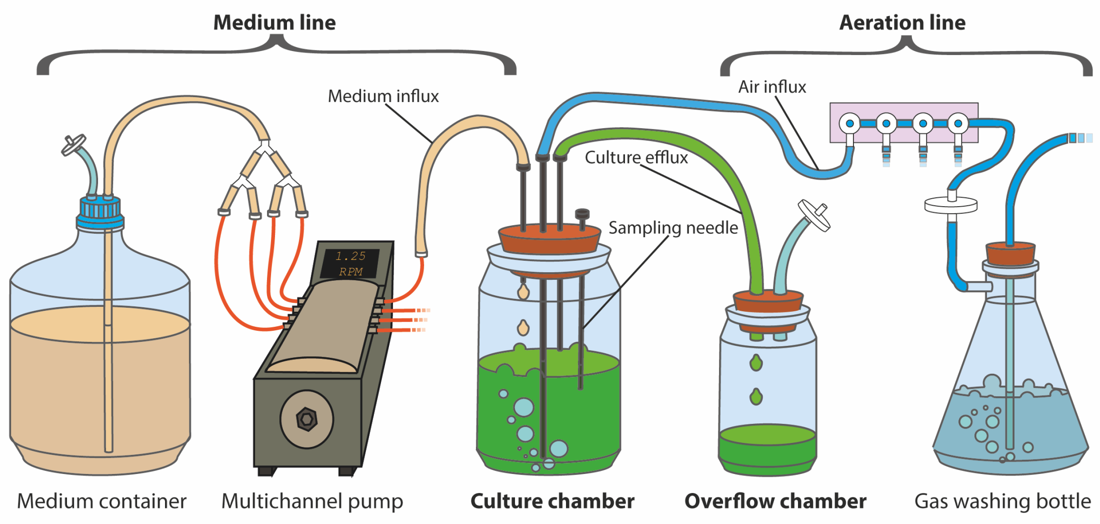

Fig 1: Simplified schematic of the mesostat system. The mesostat system includes medium container(s), a pump, culture chamber(s) with sampling needle, overflow bottle(s), a gas washing bottle along with the medium and air influx lines and the culture efflux line.

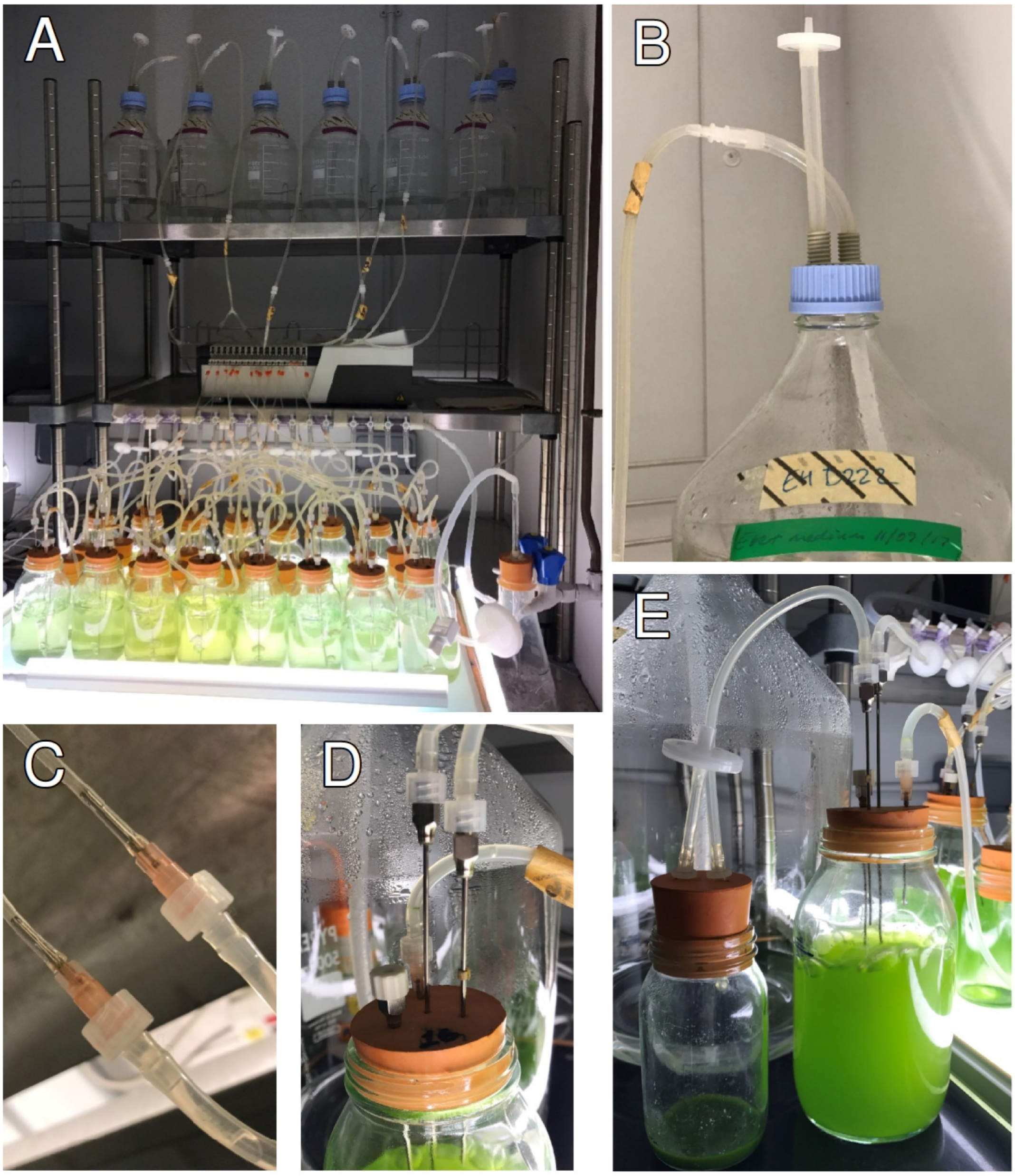

Photographs of the mesostat system. A) The complete setup just after inoculation with algae, running an experiment with six levels of treatments applied through the media lines. B) Close-up of medium siphon through medium container lid. C) Close-up of connection between pump tubing and silicone tubing used throughout array. D) Close-up of culture chambers rubber bung with the four hypodermic needles, capped sampling needle to the left in foreground, steel efflux needle to the right in foreground, steel aeration needle in the middle, and pink plastic medium influx needle in the background. E)The overflow chamber (left) and the culture chamber at steady state (right) with the efflux line running between them.

The medium containers

The volume and number of medium containers depends on the experimental design and number of treatments. When the experimental design calls for different treatments applied via the medium, culture chambers sharing a treatment share a medium source. The volume of the medium containers should be chosen to allow sufficient medium to supply all its chambers for at least 5 days, to ensure the time to prepare new sterile medium before changing is needed. The depletion rate of the medium will depend on the number of chambers sharing a container and the flow rate.

The medium container should be sealed with a lid or stopper with a hole that allows air to escape through a filter. If the medium outflow can be through either a tap at the bottom of the container, or tubing through the lid siphoning medium from the bottom. A single 20 L autoclaveable glass container, fitted with a silicone stopper and a filter (0.45 µm) on top and a tap connected to large silicone tubing (inner/wall diameter: 12.5/2.25 mm) at the bottom, is sufficient when all 16 chambers are receiving the same medium.

For data obtained from experiments where different treatment levels were present, 5 L or 2 L autoclaveable glass bottles are best used for the treatments, each fitted with medium silicone tubing (inner/wall diameter: 6.5/1.5 mm) siphoning the medium out through holes in the lids alongside tubing ending in a filter to allow air to escape the bottle (Fig 2B). For example, as seen in Fig 2A, 6 different herbicide concentrations are applied through the medium to two chambers each from 2L bottles, and the control treatment is supplied to four chambers from a 5L bottle.

The pump and the medium lines
The medium is pumped from its container(s) by a multichannel peristaltic pump.Our design employed a Watson-Marlow 205S/CA16 (16 channels) and pumped liquid through small silicone tubing (inner/wall diameter: 3/1 mm), connected to the medium container tubing with a reducing connector. The tubing is split using Y-connectors before the pump so that each culture chamber has its own media line. The same tubing is used throughout the mesostat array (Fig 2A), except for the tubing mounted in the pump itself which is autoclaveable marprene tubing (Watson-Marlow, orange/orange, 0.88 mm bore), connected to the silicone tubing using cut off and blunted hypodermic needles (18 G, 50 mm) inserted into the pump tubing and connected to the silicone tubing using male luer locks (Fig 2C).

The culture chambers
Each culture chamber consists of a 500 ml glass jar, sealed with a rubber bung. The rubber bung has four hypodermic needles inserted through it (Fig 2D): a medium influx needle (18 G, 50 mm) connected to the tubing running through the pump, an aeration needle (16G, 203 mm) connected to the aeration system allowing constant mixing of the culture, a sampling needle (16 G, 203 or 101 mm depending on sampling needs), and an efflux needle (16 G, 101 mm) which sets the culture volume.

Tubing is connected to the medium influx, aeration, and efflux needles using male luer locks. A sterile syringe is used with the sampling needle to pull samples out of the chamber or for injections, which is kept sealed with a male luer cap when not in use.

The aeration system
The culture chambers have a constant influx of air for mixing to prevent the organisms -- in our case algae -- from sedimenting, and to create pressure for the efflux of liquid so that the culture is kept at a constant volume. Air can be supplied by lab/building infrastructure, e.g. air supply taps, or by an aquarium pump with high enough pressure. The air passes through a filter (0.2 µm) into a gas washing bottle to prevent evaporation. The gas washing bottle consists of a 1L flask with a sidearm, with the incoming air being passed through a glass pipette into distilled water. The air is pushed from the gas washing bottle through 4-port manifold connectors (180° rotation), splitting the air supply to tubing for each culture chamber and passing it through a second filter (0.45 µm). This tubing connects to the aeration needle fitted in the culture chamber bung. The air supply

can be controlled using an adjustable clamp fitted to the tubing between the gas washing bottle and the manifold connector.

The overflow chambers and the efflux line

The efflux line from each culture chamber leads to an overflow chamber consisting of a 175 ml glass bottle sealed with a rubber bung (Fig 2E). The efflux tubing is connected to a hole in the rubber tubing using a female luer lock. A second female luer lock is also fitted on the opposite side of the hole on the underside of the bung, to serve as a funnel for the incoming liquid. A second hole connected with a female luer lock to tubing ending with a filter (0.45 µm) allows air to escape the collection chamber. This chamber should be emptied regularly and can be used to control that the flow rate remains equal between the culture chambers. Samples can be obtained from the overflow chamber, but the environment in the overflow chamber will be different from the culture chambers. With a low flow rate and a high temperature environment, the culture will evaporate quickly when its volume is low. The overflow chamber is also not being diluted with fresh medium, meaning the cells are no longer kept in exponential growth or connected to the aeration line, often resulting in sedimentation and stratification of the culture.

The light system

The light is provided by warm white light LED strip lights mounted around the chambers and between the two rows of chambers, as well as a DIY light box consisting of white light LED strip lights and a semi-transparent plastic top to diffuse the light. Equal light from all angles is essential to ensure even algal growth in the chambers. A light box is not necessary, but convenient and can be used for providing light to batch cultures or growth assays of subsamples.

Ebert, D. (2013). Web-guide to Daphnia Parasites: Culturing Daphnia Food.
http://evolution.unibas.ch/ebert/lab/algae.htm

Assembling the mesostats for the first time

1 Place all vessels and machinery in their intended location before cutting the tubing to ensure sufficient lengths and to minimise mistakes. See Fig 3--5 in the Guidelines for visual representation of how the parts connect, given here is a more detailed description with possible variations in design is given below. Note that all parts that will come into contact with the medium need to be sterilised before use.

2 **Prepare the medium containers and lids:**

2.1 *If using silicone stoppers:*

- *If the medium container has a tap*, the silicone stopper only needs a hole for air to escape. Drill a hole large enough to squeeze medium size tubing

through but ensuring a tight seal so that no air can escape around the edges. Attach a short piece of tubing, sticking out about 1 cm on the underside of the stopper and 5 cm on top. Attach a 0.45-micron filter to the tubing.

- *If the medium container does not have a tap*, a medium siphon is also needed. Drill a second hole in the silicone stopper of the same size and squeeze through medium size tubing long enough to reach the bottom of the container and sticking up about 5 cm on top of the stopper.

If using screwtop lids with holes, first insert a short piece of medium size tubing through one hole so that it ends just inside the lid and sticks out about 5 cm on top. Attach a 0.45-micron filter to the tubing. For the medium siphon, insert a piece of medium size tubing, long enough to reach the bottom of the container and stick out about 5 cm on top of the lid.

2.2 Attach the lid or stopper to the medium container.

- *If there is a medium siphon*, attach the long piece of tubing to a reducing connector.

- *If there is no medium siphon* and the medium container has a tap, attach a short piece of appropriate size tubing to the tap and attach this to a reducing connector.

3 **Prepare the medium lines:**

3.1 Attach a short piece of small size tubing to the other side of the reducing connector and attach it to a Y-connector. Split the medium line into as many channels as needed by connecting short (2—3 cm) pieces of small tubing and Y-connectors. Channels receiving the same treatment should share a medium source.

3.2 For each channel, attach small size tubing of sufficient length to reach the pump and end with a male luer lock.

3.3 For each channel, cut off two 18G hypodermic needles to 0.5 cm and insert them into the ends of a piece of pump tubing. Be careful to not cut up the inside of the pump tubing, if pieces detach, they will cause blockages. The ends of the needles may need additional blunting or filing to reduce sharpness. A damaged piece of pump tubing can easily be cut off.

3.4 Connect the pump tubing to the media lines by screwing the needle luer end to the male luer lock. Screw another male luer lock to the other end of the pump tubing.

3.5 Cut a piece of small size tubing long enough to reach from the pump to the culture chamber with some slack. Plan carefully where each chamber is going to sit, if multiple treatments are used they should be distributed randomly throughout the array to avoid effects of e.g. differing light level. Attach this piece of tubing to the male luer lock at the end of the pump tubing, and end with another male luer lock.

3.6 Label the tubing with autoclave tape so you know which chamber it should connect to.

4 Prepare the culture and overflow chambers:

4.1 For each chamber, prepare the culture chamber rubber bung by drilling four 1 mm holes, one for each hypodermic needle. This aids pushing the needles through, but the seal around them should still be very tight.

4.2 Push through one 18 G short needle (medium influx), one 18 G 20 cm needle (air influx) and two 18 G 10 cm needles (culture efflux and sampling needle). A longer sampling needle can be used if desired.

4.3 Label the rubber bung so you know which chamber it belongs to.

4.4 Mark the 500 ml glass jar at the desired volume (380 ml) as well as plus and minus 19 ml (i.e. plus and minus 5%) to make the magnitude of any volume inconsistencies easier to judge by eye.

4.5 Attach the media line to the medium influx needle.

4.6 Cover the sampling needle with a male luer cap.

4.7 Attach a male luer lock to the culture efflux needle and set the needle at the height required for the desired culture volume, i.e. it should be skimming the surface of the culture.

4.8 For each culture chamber, prepare an overflow chamber. For each overflow chamber, prepare a rubber bung by drilling two holes large enough to squeeze

the lock end of female luer locks into (approximately 2.5 mm in diameter).

4.9 Connect one hole with a small size of tubing to the male luer lock on the culture efflux needle, and attach another female luer lock on the underside of the bung as a funnel. Attach a short piece of small size tubing to the other hole and end with a 0.2-micron filter.

4.10 Attach a male luer lock and push the air influx needle down to touch the bottom of the chamber.
Mount lights around the chambers and/or place the chambers on a light table, using a light meter to ensure light levels are even.

5 **Prepare the aeration system:**

5.1 Mount the 4-port manifold(s) above the chambers using clamps or tape so that the aeration tubing will be held up without kinks.

5.2 Attach a short piece of small size tubing and a 0.2-micron filter to each active port on the manifold.

5.3 Connect a small size tube to the air influx needle long enough to reach the 4-port manifold and attach to the other side of the filter.

5.4 Prepare the gas washing bottle rubber bung by drilling a hole large enough to push the long glass pipette through, but tight enough that no air can escape around it. Push the pipette through down to the bottom of the flask. If needed, use a sealant around edges of the hole.

5.5 Connect the top of the glass pipette to the air supply (building supply or an aquarium pump) with appropriate size tubing.

5.6 Put dH_2O in the gas washing bottle. The water level should be so that the water sufficiently covers the air outflow from the pipette, but not so that water enters the sidearm when the air is on. Mark upper and lower water levels on the gas washing bottle.

5.7 Connect the sidearm to a 0.2-micron filter with a short piece of medium size tubing and the filter to the manifold with a longer piece of medium size tubing. A clamp can be placed on this part of the tubing to control air flow if needed.

6 **Medium container(s):**

6.1 Disconnect the reducing connector on media line between the medium container and the pump tubing.

6.2 Prepare the medium and place in the container as it should be autoclaved with the medium inside.

6.3 Place a filter in the open tubing on the medium container.

6.4 Seal tightly with autoclave bags and autoclave tape around all filters.

6.5 Autoclave at 121 (15psi) for 30 min (longer might be necessary for larger containers).

7 **Media line:**

7.1 Disconnect at male luer locks to media influx needle.

7.2 Neatly roll up each media line and secure with autoclave tape.

7.3 Place all pieces of tubing in an autoclave bag. Autoclave at 121 (15 psi) for 30 min.

8 **Culture chamber and collection chamber bungs:**

8.1 Disconnect filters from tubing to 4-port manifold, and disconnect the male luer locks to the efflux line.

8.2 Place the rubber bungs with the needles and tubing in place into autoclaveable trays.

8.3 Place the trays in autoclave bags and seal with autoclave tape, taking care to not let the needles pierce the bag.

8.4 Autoclave at 121 (15 psi) for 30 min.

9 **Culture chamber and collection chamber jars:**

9.1 These jars are not autoclaveable. Sterilise using 70% IMS and rinse with dH_2O.

Preparing mesostats for an experiment

10 Reconnect all parts after sterilising. Wear gloves washed with 70 IMS at all times, and wear eye protection when handling the hypodermic needles.

11 When the array is assembled, start the pump to fill up the chambers and turn on the aeration system. At max speed (90 RPM) the flow rate is 2.92 ml/minute, meaning it will take approximately 2 hours and 10 minutes to fill the chambers to 380 ml.

12 When the chambers are full, ensure medium levels are equal and set pump to experiment speed. Adjust the efflux needle as necessary and monitor the overflow bottles to ensure efflux is equal

13 Turn on the lights and ensure control conditions for light level, internal culture and ambient temperatures are met (see Tab 1).

Inoculating mesostats with algae

14 From a static or continuous stock culture, fill up a couple of 15 ml falcon tubes with stock solution and centrifuge at 2000 RPM for approximately 20 minutes. If there is a red layer on top of the green pellet, remove it as this is bacterial contamination. Pour out the supernatant

and mix the pellet with fresh, sterile medium. Repeat washing procedure twice.

15 Use a sterile syringe to push an equal amount of freshly washed algal cells into each chamber through the sampling needle. Note: The inoculation volume will vary depending on the amount of stock algae available, the stock density and the desired starting density for the cultures.

16 Allow cultures to reach steady state before applying experimental treatments. See below for methods for how to sample to estimate concentration.

Applying treatments to the mesostats

17 Treatments can be applied gradually through the medium line by adding the compound directly to the medium or as shock injections through the sampling needle. The two methods can also be combined. **For shock injection:**

17.1 Prepare a mixture of medium and compound at as high a concentration as practical to ensure the volume injected into the chamber is as small as possible and reducing the effects of dilution of the culture.

17.2 Before injection, remove culture at a volume corresponding to the intended injection volume by using a sterile syringe to pull it out through the sampling needle. This so the treatment can be adequately mixed into the culture, and to avoid a sudden rush of culture through the efflux line.

17.3 Inject the treatment in through the sampling needle using a sterile syringe. All chambers should receive the same injection volume, including controls.

Daily maintenance of the mesostats

18 To ensure equal conditions in all chambers, the mesostat array must be attended to daily according to the daily maintenance protocol:

18.1 Check water level in gas washing bottle is between max and min markings. Top up with distilled water if running low. Avoid going over the max marking, as this will cause water to bubble into the airflow tubing which can lead to blocked filters.

18.2 Check culture chamber airflow is satisfactory and equal. If a culture chamber has low airflow, check if the filters are blocked, first the filter on the adjoining collection chamber, then the filter connecting to the manifold, and replace if necessary. The most common cause of filter blockage is them becoming wet, either by an efflux blockage or low pressure resulting in the culture entering the aeration needling and tubing, or by high ambient humidity. Make a note of airflow problems data is being collected if it is possible the problems have been present for more than an hour. If filters are often blocked, consider changing the ambient humidity.

18.3 Check for leaks around connectors and luer locks. If leaking, first try tightening. If that does not help, replace with a new part (sometimes autoclaving can warp the luer locks), taking care to sterilise the new part with 70% IMS.

18.4 Check culture levels are even and do not deviate from the 380 ml line. If the medium level is too low, check the media influx tubing and needle for blockages. The most likely points for blockages are inside the pump tubing and any needles due to their narrow gauges. If medium influx is normal, adjust the efflux needle, and check if the level is back to normal in a couple of hours (time needed to wait dependent on flow rate and total volume deviation). If the level is too high, examine the efflux tubing and needle for blockages, along with the collection chamber filter. When unblocked, the culture chamber level should return to normal volume relatively quickly. Always make a note of culture level changes if data is being collected.

18.5 Check collection bottle levels are even, and measure volume of a few to ensure the flow rate is correct. Empty the collection bottles and note the time, so that flow rate can be calculated when the bottles are next emptied.

Sampling and population monitoring with the mesostats

19 Temporarily restrict the airflow using the adjustable clamp on the tubing. This prevents liquid from bubbling up through the sampling needle.

20 Wearing gloves washed with 70% IMS, unscrew the cap on the sampling needle and use a sterile syringe to extract liquid.

21 Put the sample in a labelled Eppendorf tube and put the cap back on the sampling needle, ensuring it is screwed on tightly. Clean up any spillage.

22 Repeat for each chamber.

23 Turn the airflow back on.

24 The samples may be stored for later processing or counting, either through flash freezing with LN_2 and subsequent storage at -80°C, or by mixing with Lugol's solution and storing in a fridge at 4°C depending on the intended use for the samples.
