# Supplementary figures and images for: Mesostats—A multiplexed, low-cost, do-it-yourself continuous culturing system for experimental evolution of mesocosms
Source: PLoS One. 2022 Jul 28;17(7):e0272052. doi: 10.1371/journal.pone.0272052 (PMC9333204; doi:10.1371/journal.pone.0272052)

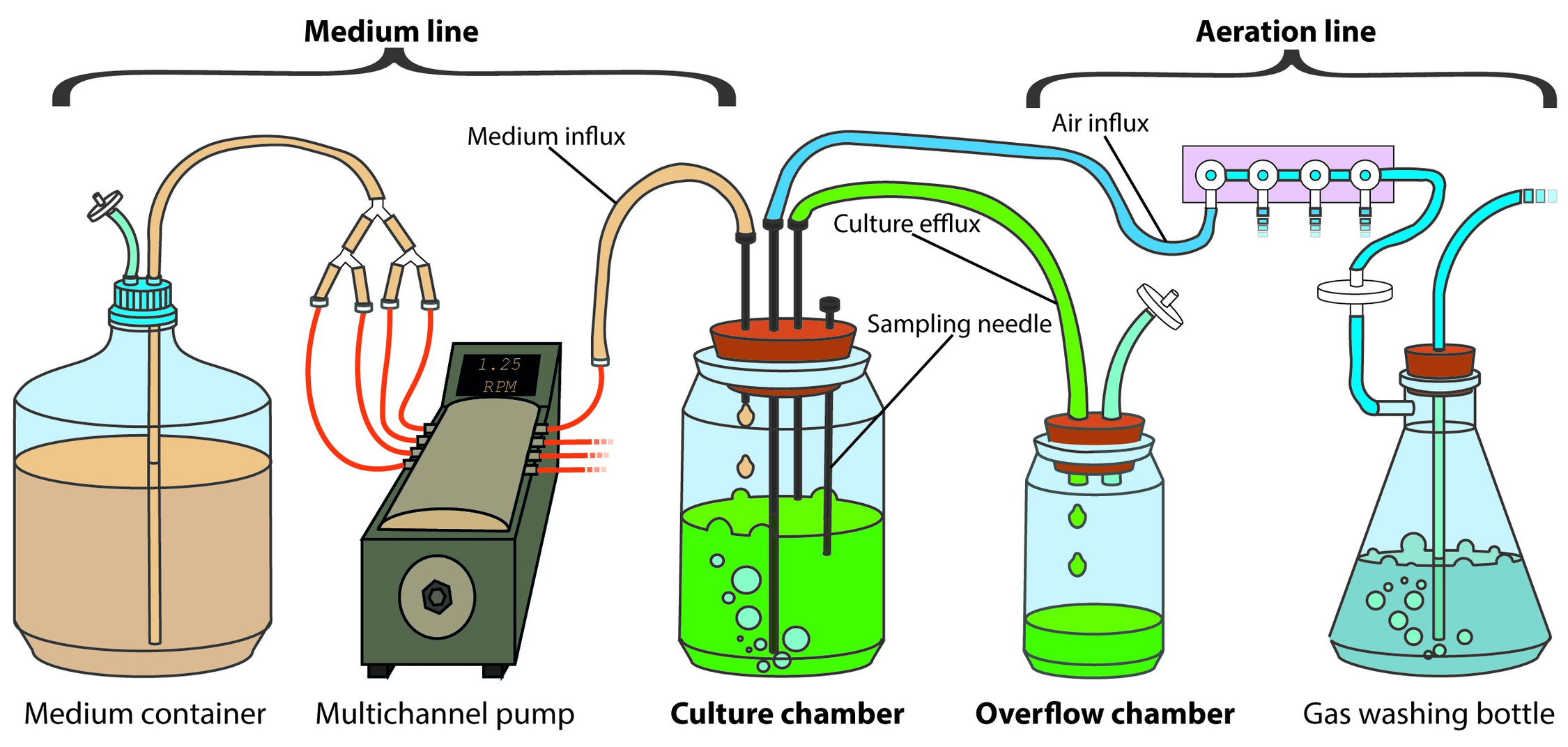

Supplement: S1 Fig — The mesostat system includes medium container(s), a pump, culture chamber(s) with sampling needle, overflow bottle(s), a gas washing bottle along with the medium and air influx lines and the culture efflux line. (TIF) [file pone.0272052.s002.tif]

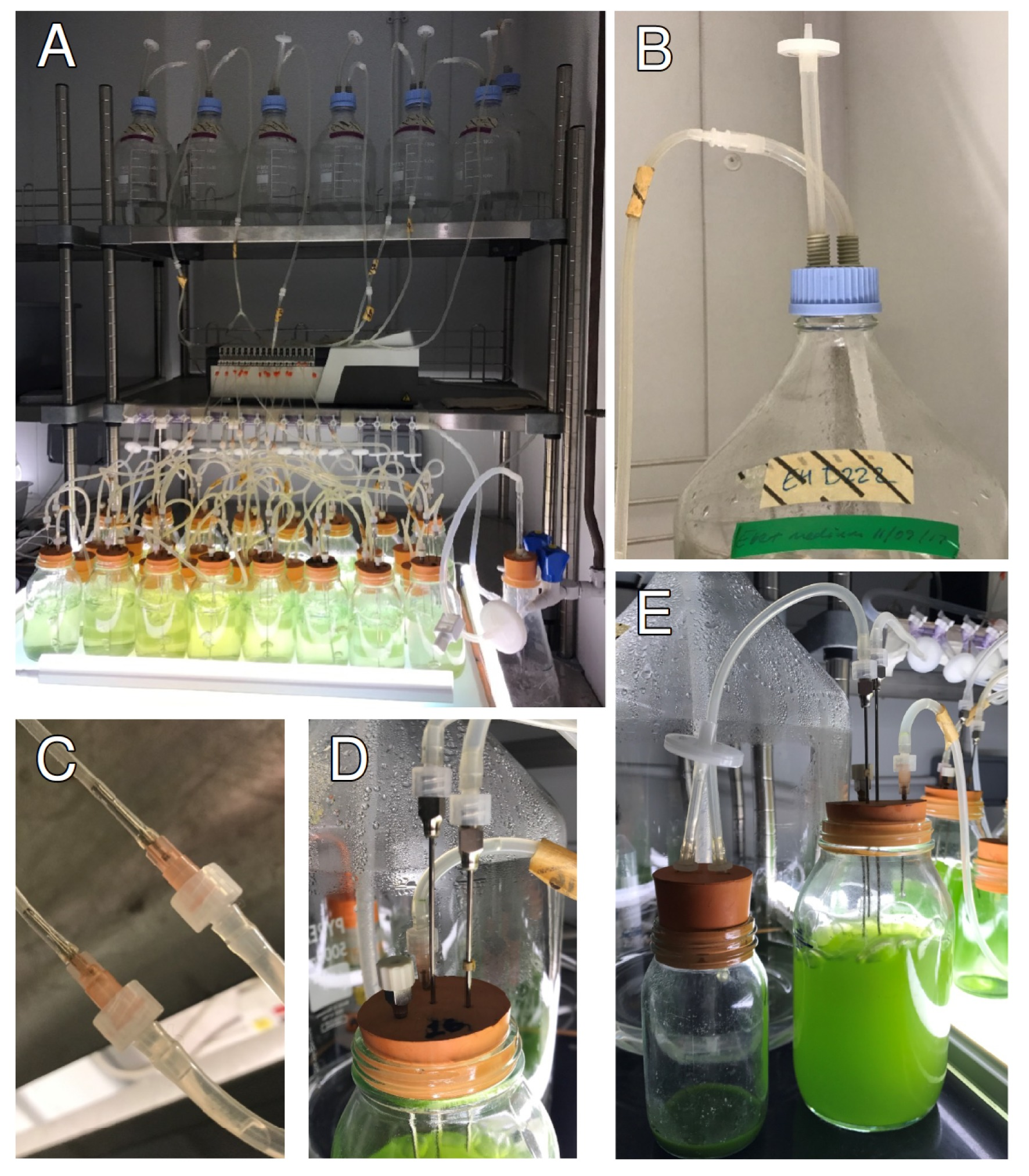

Supplement: S2 Fig — A) The complete setup just after inoculation with algae, running an experiment with six levels of treatments applied through the media lines. B) Close-up of medium siphon through medium container lid. C) Close-up of connection between pump tubing and silicone tubing used throughout array. D) Close-up of culture chambers rubber bung with the four hypodermic needles, capped sampling needle to the left in foreground, steel efflux needle to the right in foreground, steel aeration needle in the middle, and pink plastic medium influx needle in the background. E)The overflow chamber (left) and the culture chamber at steady state (right) with the efflux line running between them. (TIF) [file pone.0272052.s003.tif]

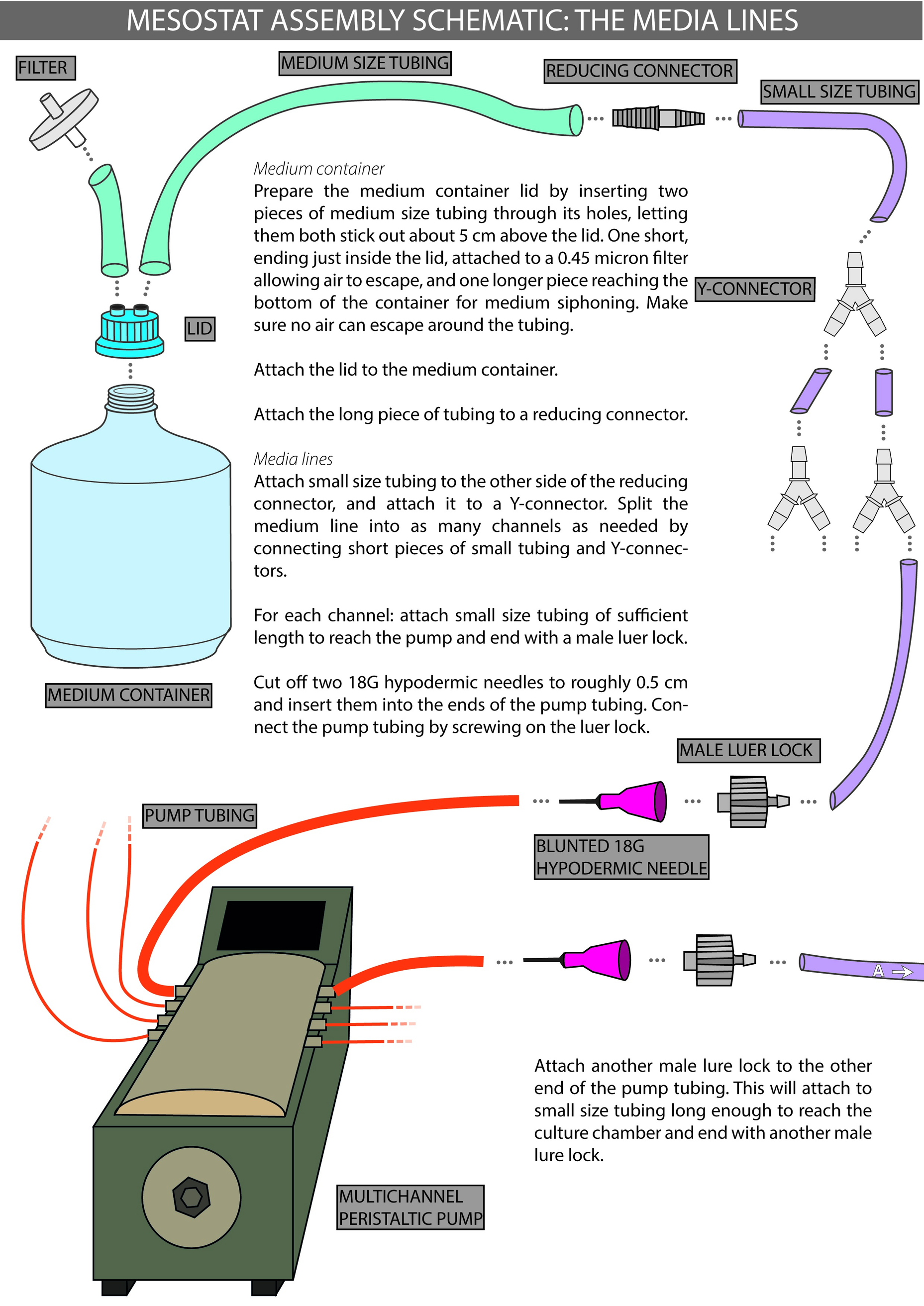

Supplement: S3 Fig — (TIF) [file pone.0272052.s004.tif]

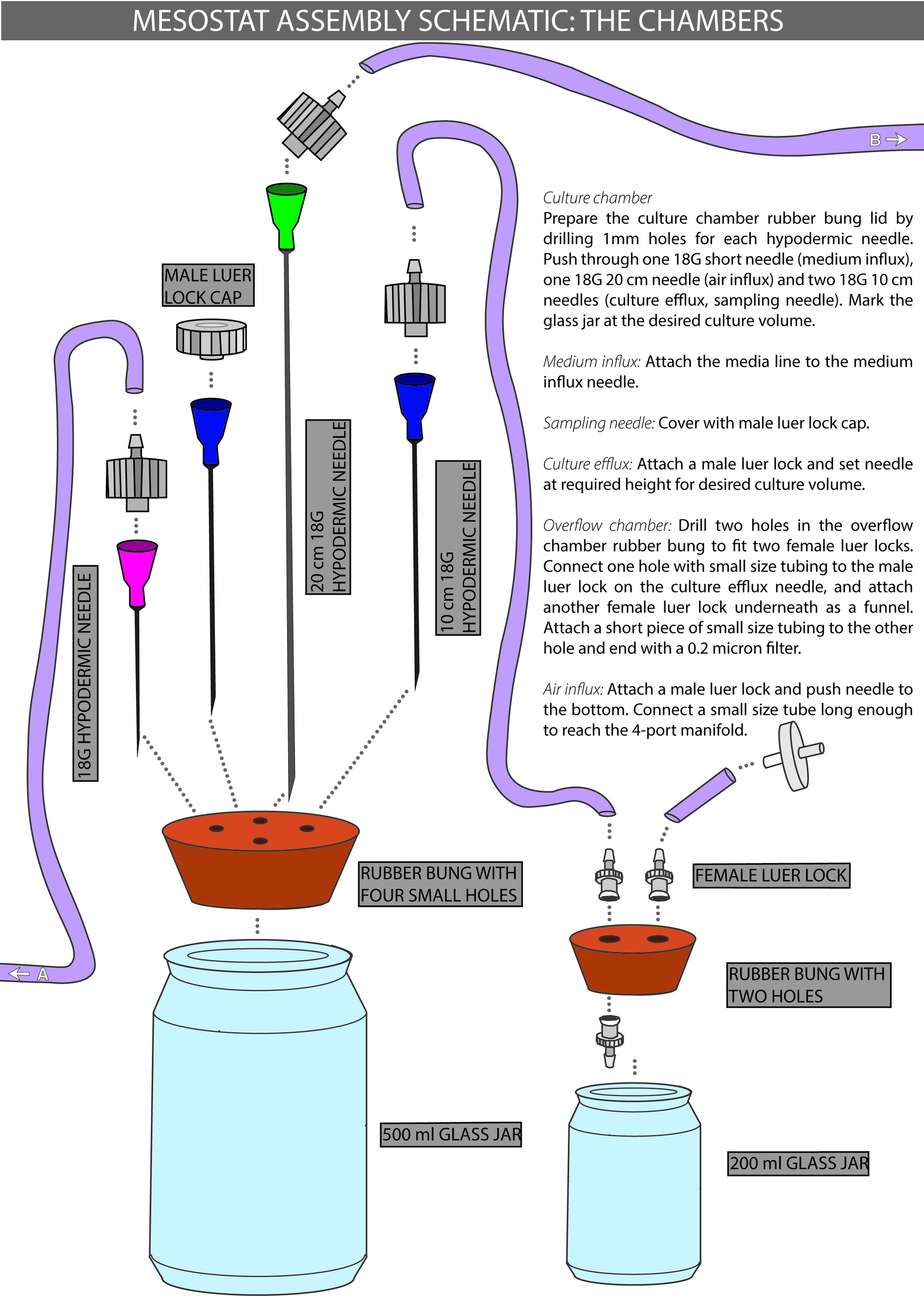

Supplement: S4 Fig — (TIF) [file pone.0272052.s005.tif]

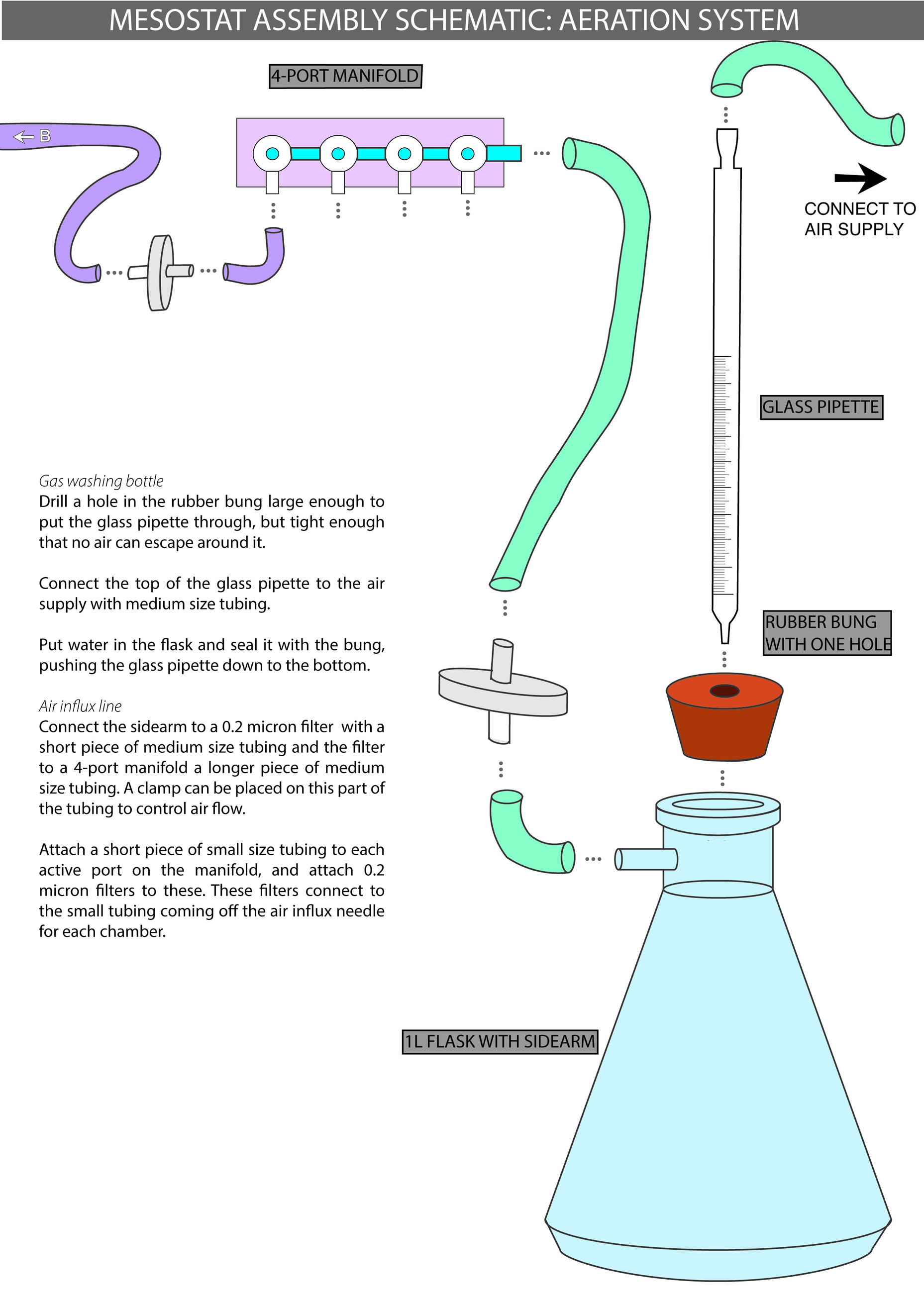

Supplement: S5 Fig — (TIF) [file pone.0272052.s006.tif]
